# Pharyngeal Complications Following Two-Jaw Surgery

**DOI:** 10.7759/cureus.76539

**Published:** 2024-12-28

**Authors:** Takafumi Yamano, Takayuki Tanaka, Shoichi Kimura, Fumitaka Omori, Kaori Wada, Yoshinobu Yokoo, Kiwako Izumi, Chinatsu Nakamichi, Mizuko Ikeda

**Affiliations:** 1 Section of Otorhinolaryngology, Department of Medicine, Fukuoka Dental College, Fukuoka, JPN; 2 Department of Otorhinolaryngology, Fukuoka Dental College Hospital, Fukuoka, JPN; 3 Section of Oral Surgery, Department of Oral and Maxillofacial Surgery, Fukuoka Dental College, Fukuoka, JPN; 4 Fukuoka College of Health Sciences, Fukuoka Dental College, Fukuoka, JPN; 5 Section of Anesthesiology, Department of Diagnostics and General Care, Fukuoka Dental College, Fukuoka, JPN

**Keywords:** laryngeal granulation, postoperative complications, tracheal intubation, two-jaw surgery, vocal cord paralysis

## Abstract

Objective: Two-jaw surgery corrects jaw deformities by adjusting occlusion and reshaping the jaw. This technique carries a high risk of pharyngolaryngeal injury due to frequent head and neck movements during intraoperative maneuvers and prolonged intubation, although the details remain unclear. This study explored the frequency and causes of postoperative pharyngeal complications following maxillary translocation.

Methodology: Between September 2019 and July 2022, 133 cases of two-jaw surgery (36 males and 97 females; mean age: 26.4 years; age range: 17-55 years) were performed in our dental and oral surgery department. Postoperatively, patients with hoarseness or pharyngeal discomfort were immediately referred to the ear, nose, and throat department to assess the pharyngeal larynx by nasal endoscopy. Patients with and without pharyngeal lesions (such as vocal cord paralysis and laryngeal granulation) were compared.

Results: The mean age, sex ratio, operative time, and blood loss were compared between groups with and without vocal cord paralysis. There was a statistically significant difference between the groups in terms of the sex ratio. No significant differences were found between groups with and without laryngeal granulation.

Conclusions: Vocal fold paralysis and laryngeal granulation were attributed to mechanical irritation of the larynx due to movement of the intubation tube during surgery, and perilaryngeal tissue compression due to hematoma and pharyngolaryngeal edema.

## Introduction

Two-jaw surgery is performed to adjust occlusion and reshape the jaw in cases of jaw deformities. Le Fort I osteotomy involves a horizontal osteotomy across the pear-shaped mouth opening and posteriorly to the winged maxillary suture, allowing the movement of maxillary fragments as a single unit. Sagittal split ramus osteotomy divides the mandibular branch laterally and medially, enabling the movement of mandibular fragments or the medial mandibular fragment as a single unit. These procedures are typically performed simultaneously [1].

This technique carries a high risk of pharyngeal injury due to the frequent head and neck movement during intraoperative maneuvers and prolonged intubation times. Although pharyngeal complications such as vocal cord paralysis [2] and laryngeal granulation [3] have been reported, the underlying mechanisms remain unclear. This study evaluated the frequency and causes of postoperative pharyngeal complications following maxillary translocation.

## Materials and methods

This study included 133 cases (36 males and 97 females; mean age: 26.4 years; range: 17-55 years) of two-jaw surgery who underwent simultaneous Le Fort I osteotomy and Sagittal split ramus osteotomy for jaw deformities performed in our dental and oral surgery department between September 2019 and July 2022. All patients were free of serious underlying diseases. Nasal intubation was performed, with intubation maintained for 24 hours postoperatively. Patients reporting hoarseness or pharyngeal discomfort were immediately referred to the ear, nose, and throat department, where their pharynx was examined via transnasal endoscopy. Patients seen in the ear, nose, and throat department were included in the study, with no exclusions. Cases with and without pharyngeal lesions (vocal cord paralysis and laryngeal granulation) were compared. Logistic regression analysis was performed for statistical analysis.

The statistical treatment was as follows. Binomial logistic regression analysis was performed with the presence or absence of vocal cord paralysis as the dependent variable and age, gender (male or female), side of nasotracheal tube insertion (left or right), operative time, and blood loss as independent variables. Similarly, a binomial logistic regression analysis was performed with the presence or absence of laryngeal granulation as the dependent variable and age, sex (male or female), side of nasotracheal tube insertion (left or right), operative time, and blood loss as independent variables. The method of variable selection was the method of increasing variables.

The statistical software was IBM SPSS Statistics version 29 (IBM Corp., Armonk, NY).

The study was approved by the Fukuoka Gakuen Research Ethics Committee (permission no. 633).

## Results

Comparison of groups with and without vocal cord paralysis

An example of pharyngolaryngoscopic findings of vocal cord paralysis is shown in Video 1, illustrating left vocal cord fixation in a paracentral position.

**Video 1:** Example of pharyngolaryngoscopic findings in a 25-year-old male with vocal cord paralysis. The left vocal folds are fixed in the paracentral position.

Table 1 presents a comparison of patients with and without vocal fold paralysis. In total, 13 cases (9.8%) exhibited vocal cord paralysis, whereas 120 cases (90.2%) did not. The mean age was 25.8 years in the group with paralysis and 26.6 years in the group without paralysis. The sex ratio was 8 males to 5 females in the paralysis group, compared to 27 males to 93 females in the non-paralysis group. The mean operative time was 246 (range: 220-550) minutes in the paralysis group and 260 (range: 135-420) minutes in the non-paralysis group. The mean blood loss was 393 (range: 220-550) mL in the paralysis group and 297 (range: 20-1230) mL in the non-paralysis group. The male-to-female ratio showed a statistically significant difference.

**Table 1:** Comparison of groups with and without vocal fold paralysis. Statistically significant differences were observed between the male and female groups (*P* < 0.01). Binomial logistic regression analysis. *P＜0.01. NS, not significant

	With vocal fold paralysis（*N*＝13）	Without vocal fold paralysis（*N*＝120）	
Percentage	9.80%	90.20%	
Mean age (range)	25.8 (17-43) years	26.6 (17-55) years	NS
Gender	８males	27 males	*
	５females	93 females	
Mean operating time (range)	246（220-550) minutes	260 (135-420) minutes	NS
Mean blood loss (range)	393 (220-550) mL	297 (20-1230) mL	NS

All cases of vocal cord paralysis were unilateral, with seven cases on the left side and six on the right. No significant differences were observed between the sides. No cricoid cartilage dislocation was observed. No specific treatment, including medication or rehabilitation, was administered, and all patients improved within two months postoperatively.

Comparison of groups with and without laryngeal granulation

Video 2 presents the representative pharyngolaryngoscopic findings of laryngeal granulation, illustrating an elevated lesion posterior to the right vocal fold mucosa.

**Video 2:** The right laryngeal granulationThe right laryngeal granulation: Pharyngolaryngoscopic findings in a 31-year-old female, demonstrating a basal elevated lesion posterior to the right vocal fold mucosa.

Table 2 presents a comparison of patients with and without laryngeal granulation. Six patients (4.5%) had laryngeal granulation, whereas 127 (95.5%) did not. The mean age was 27.8 years in the granulation group and 26.4 years in the non-granulation group. The male-to-female ratio was 1:5 in the granulation group, compared to 34 males to 93 females in the non-granulation group. The mean operative time was 297 (range: 210-420) minutes in the granulation group and 257 (range: 135-425) minutes in the non-granulation group. The mean blood loss was 420 (range: 135-550) mL in the granulation group and 301 (range: 20-1230) mL in the non-granulation group.

**Table 2:** Comparison of groups with and without laryngeal granulation. No statistically significant differences were observed among categories. Binomial logistic regression analysis. NS, not significant

	With laryngeal granulation (*N *＝ 6）	Without laryngeal granulation (*N* ＝ 127）	
Percentage	4.50%	95.50%	
Mean age (range)	27.8 (19-55) years	26.4 (17-55) years	NS
Gender	１male	34 males	NS
	５females	93 females	
Mean operating time (range)	297 (210-420) minutes	257 (135-425) minutes	NS
Mean blood loss (range)	420 (135-550) mL	301 (20-1230) mL	NS

No statistically significant differences were found across all parameters. Conservative treatment, including the use of proton pump inhibitors, led to full recovery in all cases within two to eight months.

## Discussion

Two-jaw surgery, performed for facial deformities and aesthetic purposes, can result in complications such as hemorrhage, nerve damage, neuropathic pain, nasal and temporomandibular joint disorders, and respiratory failure [4,5]. Although pharyngeal complications have been reported [2,3], their mechanisms remain unclear.

The incidence of vocal cord paralysis in cases of routine postoperative tracheal intubation is 0.03% [6], increasing to 7% with intubation durations exceeding 12 hours [7]. The risk increases threefold in patients aged >50 years, twofold if intubation duration is three to six hours, 15-fold if intubation exceeds six hours, and twofold in cases of comorbidities such as diabetes or hypertension [8]. The frequency of vocal cord paralysis in this study was 9.8%. Considering that the study participants had no underlying diseases, as well as factors such as age and operative time, the observed paralysis was deemed relatively high and specific to this technique.

Laryngeal granulomas occur in 0.01%-3.5% of cases following tracheal intubation [9]. Laryngeal mucosa ulcers or granulomas are observed in 57% of patients intubated for more than 12 hours in the intensive care unit, with associations observed for diabetes, body shape, and tube size [10]. Song et al. [3] identified factors such as frequent movement of the endotracheal tube, inadequate management in the intensive care unit (including pain control and sedation), and prolonged intubation times as contributing causes. Regarding gender differences, women have lower resistance to epiglottic trauma and a higher prevalence of post-intubation laryngeal granulomas compared to men [11]. Morphological analysis has shown changes in the dermis, including vascular proliferation, inflammation, and fibroblast intracytoplasmic alterations, indicating cellular dysfunction and damage [12]. In the present study, the participants were predominantly healthy, young women. It is likely that surgical manipulation, rather than other factors, led to contact between the tube and the laryngeal mucosa, resulting in mucosal inflammation.

Two hypotheses have been proposed to explain the causes of vocal cord paralysis and laryngeal granulation. The first hypothesis involves mechanical irritation of the vocal cords due to tube movement. A distinctive aspect of this procedure is the repeated forward bending and extension of the cervical spine during the operation to assess occlusion [3]. This maneuver likely causes significant mechanical stimulation of the larynx due to tube movement. The second hypothesis is the compression of perilaryngeal tissues caused by pharyngolaryngeal edema. After this procedure, the pharyngeal cavity becomes narrowed [13], and there have been reports of severe edema [14], sometimes leading to dyspnea. Additionally, two-jaw surgery is associated with greater blood loss than unilateral maxillary or mandibular surgery [15]. In many cases in this study, blood pooling in the pharyngeal region was observed. This suggests that hematoma formation extended into the trachea-oesophageal groove, compressing the tissue around the recurrent laryngeal nerve, which controls vocal fold mobility and the surrounding nutrient vessels, resulting in hypoperfusion and vocal cord paralysis. The predominance of males in the group with vocal cord paralysis compared to the group without paralysis is likely due to anatomical and physiological differences. Males typically have more muscle mass and a narrower cervical gap, making the recurrent laryngeal nerve more susceptible to compression.

There were several limitations of the present study. First, the study was restricted to patients who consulted an otorhinolaryngologist. This may have led to the exclusion of patients with mild subjective symptoms and those who did not seek medical attention. Second, the hypothesized causes of vocal cord paralysis and laryngeal granulation remain unproven, as postoperative computed tomography scans of the neck and chest were not performed. To address these gaps, future studies should develop standardized protocols or implement measures such as imaging studies when postoperative pharyngeal lesions are detected.

## Conclusions

Postoperative complications involving the pharynx following two-jaw surgery were analyzed. The underlying pathology is thought to involve mechanical irritation of the larynx caused by intubation tube movement during surgical maneuvers, as well as compression of perilaryngeal tissues resulting from blood pooling and pharyngolaryngeal edema. Although the prognosis for these complications is typically favorable, it is crucial to recognize pharyngolaryngeal lesions, such as vocal cord paralysis and laryngeal granulation, as potential postoperative complications.
